# Robotisch assistierte und minimal-invasive Pedikelschraubenplatzierung an der Lendenwirbelsäule

**DOI:** 10.1007/s00113-025-01596-5

**Published:** 2025-06-26

**Authors:** Dominik M. Haida, Oybek Khakimov, Stefan Huber-Wagner

**Affiliations:** 1 Technische Universität München, Klinikum rechts der Isar, Klinik für Unfallchirurgie, Ismaninger Straße 22, 81675 München, Deutschland; 2 Klinik für Unfallchirurgie, Wirbelsäulenchirurgie und Alterstraumatologie, Diak Klinikum Landkreis Schwäbisch Hall, Diakoniestraße 10, 74523 Schwäbisch Hall, Deutschland

**Keywords:** Navigation, Robotik, Wirbelsäule, Pedikelschrauben, Roboterarm, Navigation, Robotics, Spine, Pedicle screws, Robotic arm

## Abstract

**Operationsziele:**

Mit dieser Operationstechnik werden die Ziele verfolgt, die pathologische Fraktur eines Wirbelkörpers mittels einer dorsalen Spondylodese zu überbrücken, die Wirbelsäule zu stabilisieren, Schmerzen zu reduzieren und die Mobilität wiederzuerlangen.

**Indikation:**

Durch die Wirbelkörperfraktur (OF 2) des 3. Lendenwirbelkörpers (LWK 3) und die damit einhergehenden Schmerzen und Immobilität sowie auf Basis des modifizierten Scores des AO-Spine-DGOU-Osteoporotic-Fracture(OF)-Klassifikationssystems.

**Kontraindikationen:**

Keine spezifischen Kontraindikationen.

**Operationstechnik:**

Technisches Set-up: „Robotic Suite“ (Fa. Brainlab, München, Deutschland) mit Navigationseinheit „Curve Navigation System“, robotischem 3D-Cone-Beam-CT (CBCT) „Loop-X“, robotischem Arm „Cirq Arm System“ und Wandmonitor „BUZZ“.

Folgende Operationsschritte werden im Video gezeigt (Englisch):

Planung der Schrauben nach präoperativer CT-Bildgebung.

Platzieren der Patientenreferenzeinheit. Erste Bildgebung, Bildfusion und Fusionskontrolle. Registrierung der Instrumente. Robotisch assistierte Hautschnittplanung. Robotisch assistierte Bohrungen. Einbringen der K‑Drähte. Zweite Bildgebung und Kontrolle der Drahtlage. Schraubenplatzierung. Zementaugmentation der Schrauben. Einbringen des Verbindungsstabes. Wundverschluss.

**Weiterbehandlung:**

Vollbelastung, Schmerzmedikation bei Bedarf. Physiotherapeutische Behandlung zum Erlernen von Kräftigungsübungen und zur Wiederherstellung der Mobilität. Medikation mit Vitamin D_3_ und Kalzium.

Eine Metallentfernung wird nicht angestrebt.

**Evidenz:**

Operationen an der lumbalen Wirbelsäule sind mittlerweile klinische Routine in der Orthopädie und Unfallchirurgie. Sie können ohne Probleme und mit einer sehr hohen Genauigkeit im Sinne einer genauen Schraubenplatzierung durchgeführt werden.

**Video online:**

In der Online-Version dieses Beitrags (10.1007/s00113-025-01596-5) finden Sie das Video zur beschriebenen Operationstechnik (QR-Code unten).

## Abstract (en)

**Objectives of the surgery:**

The aim of this operation technique is to bridge the pathological fracture of a vertebral body by a dorsal spondylodesis, to stabilize the spine, reduce the pain and regain mobility.

**Indications:**

Due to the vertebral body fracture (OF 2) of the third lumbar vertebral body (L3) and the associated pain and immobility and due to the modified score of the AO Spine-DGOU osteoporotic fracture (OF) classification system.

**Contraindications:**

No specific contraindications.

**Surgical technique:**

Technical set-up: Robotic Suite (Brainlab, Munich, Germany) with navigation unit “curve navigation system”, robotic 3D cone beam computed tomography (CBCT) “Loop-X”, robotic arm “Cirq Arm System” and wall-mounted monitor “BUZZ”.

Operation steps are shown in the video (English):

Planning of the screws based on preoperative CT imaging.

Placement of the reference array. First imaging, image fusion and fusion control. Registration of instruments. Robotically assisted skin incision planning. Robotically assisted drilling. K-wire insertion. Second imaging and wire position check. Screw placement. Cement augmentation of the screws. Insertion of the connecting rods. Wound closure.

**Follow-up:**

Full weight bearing, pain medication if required. Physiotherapeutic treatment to learn strengthening exercises and restoration of mobility. Medication with vitamin D3 and calcium.

Metal removal is not intended.

**Evidence:**

Operations on the lumbar spine are now routine clinical practice in orthopedics and trauma surgery. They can be performed without any problems and with very good accuracy in terms of precise screw placement.

## Hintergrund

Nicht nur das alltägliche Leben wird vom stetigen Fortschritt begleitet, auch in der Chirurgie wurden in den vergangenen Jahrzehnten große technologische Fortschritte errungen. Dies zeigt sich v. a. in einer stetig wachsenden Relevanz der robotischen Chirurgie. In der Unfall- und Wirbelsäulenchirurgie ist diese wachsende Relevanz ebenso zu erkennen [5–7, 15, 19, 20]. Die über Jahre hinweg entwickelten Navigationsmethoden v. a. im Bereich der Wirbelsäule werden durch den Einsatz von robotischen Assistenzsystemen sinnvoll ergänzt und können so zu einem sehr guten operativen Ergebnis im Sinne einer hohen Genauigkeit bei der Schraubenplatzierung beitragen [4, 7, 13–15].

Mit diesem Beitrag wollen wir die Möglichkeiten einer robotisch assistierten Schraubenplatzierung an der Lendenwirbelsäule (LWS) mittels moderner Cone-Beam-CT(CBCT)-Bildgebung, Navigation und robotischer Assistenz in einem voll ausgestattetem 3D-Navigation-Hybrid-OP näher erläutern.

## Definitionen und Klassifikationen

Osteoporotische Frakturen der Wirbelsäule können nach dem validierten AO-Spine-DGOU-Osteoporotic-Fracture(OF)-Klassifikationssystem eingeteilt werden [2, 16, 17]. Dieses Klassifikationssystem teilt die Frakturen anhand ihrer Morphologie und Deformität in die Kategorien OF 1 bis OF 5 ein.

Zur Therapieentscheidung kann der noch nicht validierte, modifizierte Score des AO-Spine-DGOU-Osteoporotic-Fracture(OF)-Klassifikationssystems hinzugezogen werden [12].

## Operationsindikation

Bei für diesen Beitrag ausgewähltem Fall ergibt sich die Operationsindikation durch die osteoporotische Fraktur des 3. Lendenwirbelkörpers (OF 2), die damit einhergehenden Schmerzen (visuelle Analogskala [VAS] 6) und die resultierende progrediente Immobilität des Patienten. Dazu diente der noch nicht validierte, modifizierte Score des AO-Spine-DGOU-Osteoporotic-Fracture(OF)-Klassifikationssystems als Referenz, da nach diesem Score bei Punktwerten > 6 (Patient: 7 Pkt.) eine Operation als Therapieempfehlung genannt wird [2, 12, 16, 17].

## Fallbeschreibung

Ein 60 Jahre alter Patienten stellte sich zunächst bei seinem niedergelassenen Orthopäden mit isolierten, aber zunehmenden Schmerzen im Bereich der LWS und progredienter Immobilität aufgrund der Schmerzen vor. Diesen Schmerzen lag kein Trauma zugrunde. Vom niedergelassenen Orthopäden wurde daraufhin eine CT-Bildgebung veranlasst, durch welche eine pathologische Fraktur des 3. Lendenwirbelkörpers diagnostiziert werden konnte (OF-2-Fraktur) [2, 17]. Daraufhin suchte der Patient die Wirbelsäulensprechstunde unserer Klinik auf.

In unserer Klinik wurde die bisher erfolgte Diagnostik um eine quantitative Knochendichtemessung erweitert (T-Wert: −2,7) und der Patient über die Therapiemöglichkeiten aufgeklärt.

Gemeinsam mit dem Patienten wurde die Entscheidung zur operativen Versorgung getroffen. Dies geschah mit den Zielen der Stabilisierung, Schmerzreduktion und des Wiedererlangens der Mobilität des Patienten.

## Operationstechnik

Dorsale Stabilisierungen und die damit einhergehende Platzierung von Pedikelschrauben werden in unserer Klinik regelmäßig robotisch assistiert durchgeführt. Die Operationen werden in einem modernen Hybrid-OP, der „Robotic Suite“, durchgeführt.

Diese besteht aus der Navigationseinheit „Curve Navigation System“, aus dem robotischen Cone beam CT (CBCT) „Loop-X“, dem Wandmonitor „BUZZ“, dem robotischen Arm „Cirq Arm System“ sowie Mixed-Reality-Brillen zur Planung (Brainlab, München, Deutschland).

Bei diesem Eingriff befindet sich der Patient in Bauchlage auf dem Carbontisch, sodass die Beine des Patienten in Richtung des CBCT zeigen.

Als Instrumentarium wurde das System „Solera“ der Fa. Medtronic (Meerbusch, Deutschland) verwendet. Für die Osteosynthese wurden 4 Schrauben aus diesem System mit den Maßen 7,5 × 55 mm verwendet.

Vor der Schraubenplanung wird zunächst eine detaillierte Computertomographie (CT) Aufnahme der Lendenwirbelsäule angefertigt (0:08 min). Auf Basis dieser Aufnahme wird nachfolgend die Schraubenplanung durchgeführt (0:17 min). In dem hier dargestellten Fall, einer LWK-3-Fraktur, wurde die dorsale Stabilisierung vom 2. LWK auf den 4. LWK geplant. Dabei wurden die 4 Pedikelschrauben für die dorsale Stabilisierung und die Verbindung mittels Stabs geplant. Bereits vor der Operation erfolgte der Entschluss zur Zementaugmentation der 4 Pedikelschrauben aufgrund der bereits auch in den nichtfrakturierten Wirbelkörpern vermuteten und schließlich auch vorherrschenden verminderten Knochenqualität.

Als erster Schritt erfolgt die Befestigung der Patientenreferenzeinheit mittels einer Carbonklemme auf dem Dornfortsatz des 1. Lendenwirbelkörpers (0:29 min). Neben der Sichtbarkeit der Referenzeinheit während der Navigation sollte zudem ein möglichst geringer Abstand zwischen dem Eingriffsbereich und der Referenzeinheit zur Erlangung einer möglichst hohen Genauigkeit herrschen.

**Abb. 1:**
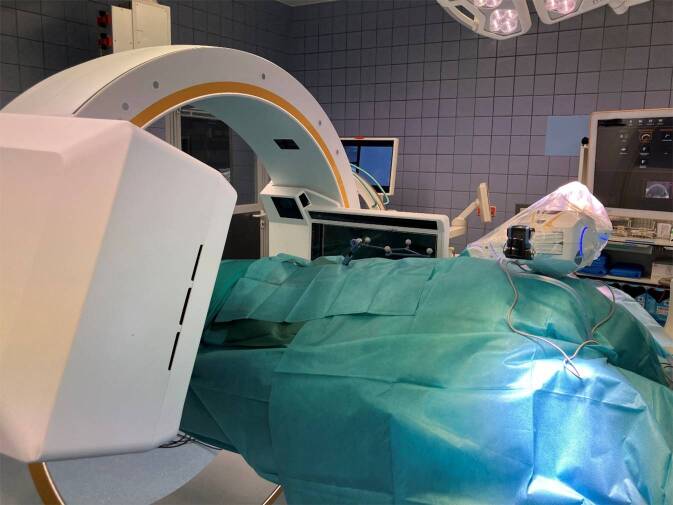
Technisches Set-up unmittelbar vor dem Scan

Als nächster Schritt folgt die Bildgebung mittels CBCT. Nach dem Abdecken des Eingriffsbereiches mittels eines Schlitztuches (0:45 min) und dem Einfahren des CBCT (0:47 min) folgt die Feldaufnahme zur Kontrolle der Abbildbarkeit des Eingriffsbereiches (0:53 min). Unmittelbar vor dem Scan erfolgt dann die Kollisionsüberprüfung (0:57 min). Die Speicherung der Position von Tisch und CBCT sollte nun für die nachfolgenden Scans erfolgen. Nach der Durchführung dieser vorbereitenden Schritte kann der eigentliche Scan durchgeführt werden (1:09 min; Abb. 1).

Auf die Bildgebung folgt die Fusion des präoperativ durchgeführten CT mit der enthaltenen Schraubenplanung mit dem intraoperativen CT (1:19 min). Zur Kontrolle der korrekten Fusion sollten eine visuelle Kontrolle des Fusionsergebnisses auf dem Bildschirm anhand anatomischer Landmarken wie auch eine Kontrolle mittels Pointer am Patienten durchgeführt werden. Wurde sich des erfolgreichen Fusionsergebnisses vergewissert, können die zu verwendenden Instrumente im System registriert werden (1:53 min).

**Abb. 2:**
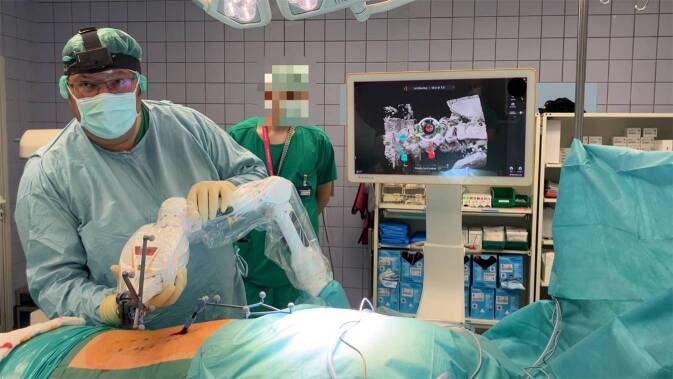
Positionieren des Roboterarms vor der Anfahrt an das geplante Bohrtrajekt

Im nächsten Schritt folgt die robotisch assistierte und minimal-invasive Hautschnittplanung (1:59 min). Der Roboterarm wird mit Blick auf den Bildschirm zum geplanten Trajekt geführt und so eingestellt, dass das geplante Trajekt auf dem Bildschirm grün erscheint (Abb. 2). Nun kann der Roboterarm losgelassen werden, dieser stellt sich daraufhin auf das geplante Trajekt ein. Leuchtet der Roboter grün, befindet er sich an der Zielposition für die geplante Schraube. Mit dem Einstecken der Bohrhülse am Roboterarm kann der geplante Hautschnitt auf der Haut des Patienten angezeichnet werden.

Nach der Markierung aller Hautschnitte erfolgen die minimal-invasiven Hautschnitte (2:43 min).

Es erfolgen nun die Bohrungen für die Drähte und die anschließende Platzierung der Pedikelschrauben (2:53 min). Die Bedienung des Roboterarms erfolgt analog zur Bedienung des Roboterarms bei der robotisch assistierten Hautschnittplanung. Zum Einbringen der Bohrhülse sollten diese und die Carbonführung am Roboterarm mit Wasser angefeuchtet werden, um ein gutes Einschieben ohne Manipulation am Roboterarm zu erreichen. Die Bohrhülse kann mit einer Schere durch das Weichteilgewebe zum Wirbelkörper geführt werden. Dann erfolgt die vertikale Fixierung der Bohrhülse in der Carbonführung am Roboterarm mittels Umlegens eines Clips. Bei der Platzierung der Bohrhülse auf dem Wirbelkörper sollte sich der Operateur vergewissern, dass die Bohrhülse einen guten Halt findet und ein Abrutschen auf Strukturen wie den Facettengelenken ausgeschlossen wird. Deshalb kann es nötig sein, noch minimale manuelle Ausrichtungen an der Bohrhülse vorzunehmen, um genau die Richtung des geplanten Trajekts zu erreichen. Nach dem leichten Festklopfen der Bohrhülse, welche diese gegen Abrutschen sichern soll, wird diese mit einer Hand fixiert, während mit der anderen Hand die Bohrung unter Blickkontrolle auf den Bildschirm durchgeführt wird. Nach der Bohrung werden Kirschner Drähte (K‑Drähte) in die Bohrlöcher eingeführt.

Nach dem Einbringen aller K‑Drähte erfolgt der zweite CBCT-Scan (5:10 min). Die Kontrolle der Drahtlage erfolgt visuell auf dem Navigationsbildschirm (5:21 min), die Drahtlage kann dabei exakt in mehreren Ebenen gleichzeitig eingestellt und kontrolliert nachfolgt werden.

Wenn sich die Drahtlage als zufriedenstellend darstellt, kann im nächsten Schritt das Einbringen der Schrauben stattfinden (5:45 min). Ist dies geschehen, kann nachfolgend die Zementaugmentation unter C‑Bogen-Durchleuchtung durchgeführt werden (5:50 min). Nach der erfolgreichen Zementaugmentation werden die Schrauben mittels Verbindungsstab verbunden (5:54 min).

Abschließend erfolgt der sterile Wundverschluss mittels Naht und Klammern (5:57 min).

**Abb. 3:**
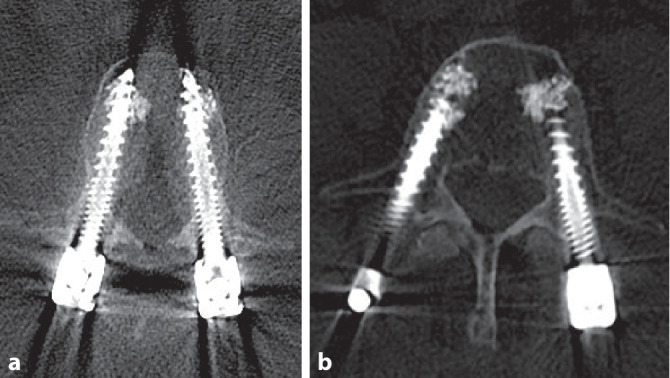
Postoperatives Ergebnis der Pedikelschraubenplatzierung. **a** LWK 2, **b** LWK 4

Postoperativ wurden konventionelle Röntgenaufnahmen und eine CT-Bildgebung des Patienten durchgeführt (6:01 min; Abb. 3), in welcher sich die Schraubenlage als sehr zufriedenstellend darstellt.

## Postoperative Behandlung

Aus der erfolgreichen Durchführung der dorsalen Stabilisierung in dem hier beschriebenen Fall resultierte die sofortige Belastbarkeit des Patienten.

Eine physiotherapeutische Weiterbehandlung zum Erlernen von Kräftigungsübungen und zur schnellen Wiedererlangung der Mobilität wurde initiiert.

Dazu wurden dem Patienten eine an die Osteoporose angepasste Medikation mit Vitamin D_3_ (2000 IE/Tag) und Kalzium (1000 mg/Tag) verordnet.

Es wurden postoperativ eine konventionelle Röntgenkontrolle sowie eine CT-Bildgebung der LWS durchgeführt.

Der Patient wurde am 7. postoperativen Tag bei Mobilität ohne Hilfsmittel in die ambulante Weiterbehandlung entlassen.

Eine Metallentfernung wird nicht angestrebt.

## Fehler, Gefahren und Komplikationen

Allgemeine chirurgische Risiken wie Wundinfektionen oder Wundheilungsstörungen sind auch bei dieser Operationstechnik gegeben.

Zusammenfassend lässt sich sagen, dass v. a. auf eine gute Schulung des OP-Teams und des Ärzteteams im Umgang und mit der Bedienung der technischen Gerätschaften zu achten ist [6, 7]. Durch die gute Schulung können schon viele potenziellen Fehlerquellen eliminiert werden, was zu einem guten und genauen Operationsergebnis beiträgt. Für eine möglichst hohe Genauigkeit sind eine entsprechend gute Planung und ebenso intraoperativ eine sehr genaue Fusion essenziell. Die Kontrolle des Fusionsergebnisses kann bzw. sollte unbedingt visuell auf dem Bildschirm und/oder haptisch mittels Pointer unter der Zuhilfenahme von anatomischen Landmarken erfolgen [6, 7].

## Evidenz der Technik

Navigierte Techniken (v. a. die Handnavigation) finden schon seit einiger Zeit in der Unfall- und Wirbelsäulenchirurgie Verwendung [8, 11, 18]. Die hierbei erreichte Genauigkeit stellt sich als sehr hoch dar. Durch die Verbindung der Navigation mit der Robotik können ebenso sehr hohe Genauigkeiten und gute Ergebnisse erreicht werden [1, 7, 9, 10, 15, 20]. Dabei lassen sich potenziell noch mehr menschliche Fehler eliminieren als bei rein navigierten Techniken. Genauere Vergleiche der Genauigkeiten und Operationsergebnisse der rein navigierten Techniken mit navigierten und robotisch assistierten Techniken müssen allerdings noch durch weitere wissenschaftliche Arbeiten quantifiziert werden.

Wir konnten im klinischen Alltag eine Genauigkeit von 98,7 % (Grade A + B in der Klassifikation von Gertzbein und Robbins) bei der Platzierung von Pedikelschrauben an der Wirbelsäule erreichen [1, 3, 7].

## Fazit für die Praxis

- Die Einteilung von osteoporotischen Frakturen der Wirbelsäule erfolgt nach der AO-Spine-DGOU-OF-Klassifikation.
- Zu Therapieentscheidungen kann der modifizierte Score der AO-Spine-DGOU-OF-Klassifikation hinzugezogen werden.
- Eine gute Schulung des an der Operation beteiligten Personals ist essenziell für einen reibungslosen Operationsablauf in einem Hybrid-OP.
- Ein Eingriff in einem Hybrid-OP mit moderner Bildgebung ermöglicht es, die Lage des Drahtes intraoperativ zu überprüfen und damit für eine sichere und genaue Schraubenplatzierung zu sorgen.
- Die erreichten Genauigkeiten von robotisch assistierten Schraubenplatzierungen sind sehr hoch.
- Eingriffe an der lumbalen Wirbelsäule können problemlos in einem 3D-Navigation-Hybrid-OP durchgeführt werden.

## Supplementary Information

Operationstechnik der robotisch assistierten und minimal-invasiven Pedikelschraubenplatzierung an der LWS
